# Novel 3D Scanning and Multi-Angle Analysis Uncover the Ontogenetic Developmental Dynamics of the Skull in *Vespertilio sinensis*

**DOI:** 10.3390/biology14101389

**Published:** 2025-10-11

**Authors:** Xintong Li, Mingyue Bao, Yang Chang, Hui Wang, Jiang Feng

**Affiliations:** 1 College of Life Science, Jilin Agricultural University, Changchun 130118, China; lixintong@mails.jlau.edu.cn (X.L.);; 2 Jilin Provincial International Cooperation Key Laboratory for Biological Control of Agricultural Pests, Changchun 130118, China; 3 College of Life Science, Northeast Agricultural University, Harbin 150030, China; 4 Jilin Provincial Key Laboratory of Animal Resource Conservation and Utilization, Northeast Normal University, Changchun 130117, China

**Keywords:** bats, developmental dynamics, morphological structure, skull, three-dimensional scanning

## Abstract

The mammalian skull, which surrounds and protects the brain, is one of the most morphologically diverse and functionally important structures in the vertebrate body. As one of the most ecologically diverse mammals, the developmental dynamics of morphological and structural changes and functional diversity in the skull of bats need to be revealed. Here, we focused on the developmental characteristics of the *Vespertilio sinensis* skull, and used statistical analysis, spatial morphology visualization, and comparative analysis of the Stretch Factors (SF) of the masticatory muscles to better understand the connection between the morphology of the skull and the development of the body size during the developmental process of *V. sinensis*, the changes in the three-dimensional (3D) spatial morphology and structure, and the correlations between opening capacity and the transformation of feeding habits. This study not only provides a new perspective for understanding the morphological adaptive mechanism of ecological niche expansion that accompanies the transition of mammalian skulls from juvenile to adult feeding but also provides a crucial scientific basis for an in-depth understanding of the growth and developmental mechanism of bats’ skull and even vertebrates as a whole, which is potentially useful for the development of ecological conservation and evolutionary biology.

## Simple Summary

Mammalian skulls, commonly used for species identification, are among the most morphologically diverse and functionally important structures. Their fine anatomical details are crucial for distinguishing species. Bat skulls play key roles in taxonomy, echolocation, ecological adaptation, and feeding habits. However, limited studies have explored how bat skull morphology adapts during growth and development. In this study, we investigated the developmental dynamics of the skull of the echolocating bat *Vespertilio sinensis* across different developmental periods. We employed a combination of field observations, three-dimensional (3D) scanning, traditional morphological measurements, spatial morphology comparisons, and the construction of a model of the Stretch Factors (SF) of the superficial masticatory muscles. Our findings reveal the intrinsic correlations between skull morphology and various developmental aspects, including body size development, changes in 3D spatial morphology and structure, jaw opening capacity, and shifts in feeding habits. Our findings address the deficiency in the understanding of bat skull development and shed light on the morphological adaptive mechanisms underlying the transition from juvenile to adult feeding behaviors and ecological niche expansion in mammals.

## 1. Introduction

Ecomorphology assumes that morphological changes in animals are related to behavior, performance, and fitness, and that they are influenced by heredity, living environment, and feeding habits [1,2,3,4,5]. The skull is one of the most morphologically diverse and functionally important structures in the vertebrate body [6]. The mammalian skull is an informative and versatile study system critical to research efforts across the broad spectrum of molecular, cellular, organismal, developmental, and evolutionary sciences. The mammalian skull, an anatomical entity and a well-distinguishable morphological unit, is one of the best examples of how modern morphological research intercalates with other fields of biology and how discoveries from these other disciplines enhance and fulfill one another [7].

Previously, skull morphology was often used for species delimitation and identification [8], and its fine anatomical details are important key characters in discriminating species [9]. Bats (Order Chiroptera) are one of the most ecologically diverse mammals [6]. Detailed work has been conducted on the skull development in Chiroptera [10]; however, most studies on cranial morphology are focused on taxonomically useful characters [11,12,13]. The skull of insectivorous bats functions in capturing, subduing, and handling prey, as well as defending against predators [14,15,16]. Across bat lineages, the skull universally harbors high morphological variation and contains structures linked to vision, hearing, and smell. These sensory structures facilitate access to external information and support key physiological processes, including food collection and processing, water consumption, vocalization, and respiration [17,18,19]. This morphological and functional complexity enables linking trophic niche-related natural selection pressures to skull morphology [20,21]. Specifically, skull traits can predict key ecological indicators—including food composition, dietary ecotype width [22,23], and acoustic wave type [24]—as well as phylogenetic relationships [25], which reflect evolutionary affinities. With advancing technological methods, Fawcett [26] and Frick [27] used prenatal sections to describe the development of the chondrocranium in *Myotis* sp. and *Myotis myotis*, respectively. Camacho [28] presented the first 3D geometric morphometric (GM) analysis of skull shape patterns in evolution and skeletal development to determine the mechanism of cranial evolution in Phyllostomatidae bats. Ikeda [9] performed a comprehensive comparative study of *Rhinolophus ferrumequinum* complex skull morphology combining traditional linear measurements, geometric morphometric methods, and evaluation of non-quantitative discriminant characters. In addition, there are studies that used 3D geometry of the skulls as well as bite force and diet measurements to investigate the characteristics and the correlation of skull morphology and bite force to bat diets in a phylogenetic context [29].

The skull is a critical structure that reflects both individual growth and physiological development in bats. The morphological and functional diversity in adult bats’ skull remains largely unexplored for most species [30]. The skull’s adaptation to various factors during growth, including the transition from a liquid milk diet to a solid diet and the onset of independent flight, is closely linked to its postnatal development [31]. These adaptations likely drive changes in skull morphology and biting force. During development, the skull undergoes significant changes to accommodate shifts in diet and behavior. However, detailed studies on skull development are rare, with only a few species like *Eptesicus fuscus* having been comprehensively studied [32]. Additionally, the onset of flight necessitates a lightweight yet robust skull structure to support sensory systems and withstand aerodynamic forces. Given the skull’s multifaceted role in the individual development and physiological maturation of bats, detailed studies on skull development are essential for a comprehensive understanding of their life history strategies. The skull’s development can also indicate overall health and environmental pressures, making it a valuable indicator of physiological maturation. Given its importance, detailed studies on skull development are crucial for understanding bats’ adaptive mechanisms and ecological success. Such research can provide valuable insights into the adaptive mechanisms that enable bats to thrive in diverse ecological niches and contribute to the broader field of evolutionary biology.

Herein, we examined the ontogenetic morphological changes in the skulls of *Vespertilio sinensis* from the same population. *Vespertilio sinensis*, commonly known as the Asian particolored bat, belongs to the family Vespertilionidae within the order Chiroptera. These bats are widely distributed across central and eastern China, Korea, Japan, and Russia [33]. They typically roost in the roofs or eaves of bridge holes and old buildings [34]. The average weight and forearm length of adult bats are 17.41 ± 9.84 g and 49.81 ± 1.91 mm, respectively. Based on the developmental dynamics and dietary shifts in *V. sinensis* [35], we conducted 3D scans of female *V. sinensis* skulls from birth to adulthood. We measured and statistically analyzed various morphological parameters to quantify skull growth changes. This provided a crucial basis for understanding the overall growth and development of *V. sinensis*. By digitally converting the morphology using 3D images, we compared spatial morphological changes and analyzed the differential skull changes across different developmental periods. Additionally, we constructed a model of the Stretch Factors (SF) of the superficial masseter muscle to calculate the strain coefficients of the temporal and masseter muscles. This clarified the quantitative relationship between dietary shifts and skull morphology changes, revealing how *V. sinensis* adapts to different food resources through structural modifications. Our findings systematically elucidate the skull developmental dynamics of *V. sinensis* and uncover the progressive adaptive strategy of bat skull morphology with growth. This study offers a new research paradigm for mammalian developmental biology.

## 2. Materials and Methods

### 2.1. Specimen Acquisition

In this study, individuals of *V. sinensis* were collected from June to August 2023 under the Acheng District overpass in Harbin City, Heilongjiang Province, China. During the summer, female *V. sinensis* form large maternal colonies, comprising hundreds to thousands of individuals, and typically give birth to twins b etween late June and early July [36]. These female groups remain at the roosting site to nurse their young until the offspring reach near-adult size in a few months, after which they migrate with their mothers. Based on the age estimation equation proposed by Jin et al. [37], we collected bat samples across six consecutive postnatal developmental periods (each at 7-day intervals) and one adult period. This sampling strategy allowed us to capture the ontogenetic changes in skull morphology across the entire developmental spectrum of *V. sinensis* (Table 1).

For each captured bat, we recorded its body surface temperature using a Fluke 62 MAX IR thermometer (Fluke, Everett, WA, USA). Subsequently, we measured the body mass (BM) with an electronic balance (Ohaus LS 200, Ohaus, NJ, USA, precision 0.01 g) and the forearm length and epiphyseal gap with a digital caliper (TESA-CAL IP67, Tesa Technology, Renens, Switzerland, precision 0.01 mm). These measurements were used to estimate individual age [37]. For specimen preparation, only non-lactating females were selected. Additionally, we measured several basic morphological parameters of the bats, including head length, head width, head height, and head-body length, to support subsequent analyses.

### 2.2. Three-Dimensional Skull Morphology Data

We brought field-collected bat samples back to the laboratory for skull preparation, using a modified boiling method optimized for small mammal skulls to preserve delicate structures. Specifically, skulls were boiled in a 2% solution of soda water (Sodium carbonate, Na_2_CO_3_) for 10–15 min [38]. This concentration of sodium carbonate gently loosens soft tissues (muscles, connective tissues) without corroding bone or causing deformation of fragile structures. After boiling, soft tissues were manually removed with fine forceps to avoid mechanical damage to skull microstructures. Post-preparation inspections confirmed no observable deformation or damage to anatomical features critical for subsequent morphometric measurements. We set up three biological replicates (three specimens) per period, yielding 21 intact skull specimens for scanning and morphological analysis. This method was selected for two key reasons: sodium carbonate-facilitated boiling efficiently removes soft tissues while preserving bone integrity; it avoids the structural warping risk associated with prolonged boiling (without sodium carbonate) [38,39].

**Figure 1:**
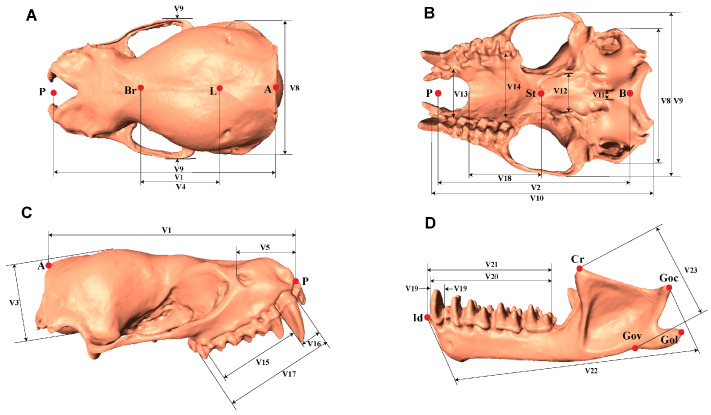
Dorsal (**A**), ventral (**B**), lateral (**C**) views of the skull (without mandible); Lateral (**D**) views of mandible displaying craniodental measurements. Detailed descriptions of each metric are in the Supplementary Information.

We used a Blu-ray 3D scanner (OKIO-5 M-100, TianYuan, Beijing, China) for skull scanning, with technical parameters as follows: single scan time < 1.5 s, resolution of 5 million pixels, scanning accuracy of 0.005 mm, and sampling point distance of 0.04 mm. This scanner features one-touch automatic feature point recognition and advanced automatic stitching technology; post-scan directional data were spliced using Geomagic Control X 2022.0.0 (3D Systems, Inc., Rock Hill, SC, USA) to generate complete 3D skull images [40]. We chose multi-angle analysis primarily because the thin-walled structures of *V. sinensis* skulls have complex surface geometries and narrow internal gaps. Single-angle scanning would create blind spots in these regions, whereas multi-angle scanning captures comprehensive 3D information—ensuring accurate reconstruction of key anatomical features (e.g., landmark points on the zygomatic arch and mandibular condylar process) required for subsequent morphometric analysis. We referred to the morphology and skull measurement methods of bats as described by Bates [41,42] and Yang et al. [43], and used *Materialise Magics* 25.0 (Materialise, Leuven, Belgium) to measure the 3D skull data obtained after scanning (Figure 1 and Figure S1).

**Figure 2:**
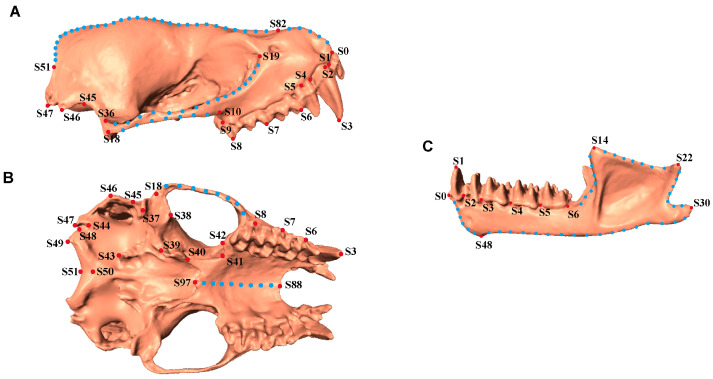
Illustration of Landmarks used for skull measurement. (**A**). Lateral view of skull (without mandible). (**B**). Ventral view of skull (without mandible). (**C**). Mandible. Red dots indicate fixed landmarks and blue dots indicate semi-landmarks on the curves. Detailed descriptions of each point are in the Supplementary Information.

According to the 3D geometric morphometric method, a total of 98 points were selected for the skull (without mandible): 31 landmark points and 67 semi-landmark points on the curve, while for the mandible, 55 points were selected: 11 landmark points and 44 semi-landmark points on the curve. The selected landmark and semi-landmark points were determined based on previous studies of skull landmarks to quantify cranial morphology. The landmarks were digitized using Landmark Editor 3.6 [44]. Each skull was digitized three times, and the average value was used for subsequent analysis. To avoid redundant information related to symmetrical skull structure, we selected the right side of the cranium and mandible for analysis (Figure 2 and Figure S2). We analyzed the shape coordinates of the skull and mandible as independent datasets for subsequent analysis.

For damaged and incomplete skulls, we estimated the location of missing landmarks using the function “*estimate.missing*” in package “geomorph 4.0.8” [45] in R 4.4.1 [46]. The missing landmarks in the incomplete specimens were designated by NA in place of the *x,y,z* coordinates. We then used the thin-plate spline (method = “*TPS*”) to interpolate landmarks on a reference specimen to estimate the locations of missing landmarks on a target specimen. Then, we performed a Generalized Procrustes analysis (GPA) for all specimens’ landmark coordinates with the function “*gpagen*” in package “geomorph 4.0.8” [45] in R 4.4.1 [46]. The landmarks and semi-landmarks were superimposed in a common coordinate system to remove the effect of location, orientation, and scale among various samples. Here, the Procrustes distance was used to optimize the semi-landmark positions along the curve to obtain the Procrustes-aligned coordinates and centroid size [47,48]. Here, Centroid Size refers to the square root of the sum of squared distances from each landmark to the centroid of the landmark configuration, and is used as a proxy for overall skull size [49]. After all specimens’ coordinates had been superimposed using GPA, we obtained the mean shape of each individual’s skull from the global dataset using the function “*mshape*” in the R package “geomorph 4.0.8” [45] (R 4.4.1 [46]) for subsequent analysis.

### 2.3. Statistical Analysis

The measured data were organized and statistically analyzed using SPSS 29.0 statistical software. Correlation analysis was used to determine the correlation between the skull parameters and basic morphological parameters of *V. sinensis* to reflect the relationship between their concomitant changes. To clarify whether there are differences in the skull parameters of *V. sinensis* at different developmental periods, 29 indicators of skull data were extracted for principal component analysis (PCA), and scatter plots were drawn based on the scores of the first and second principal components. Based on the 29 skull parameters, we computed a Euclidean distance matrix using the “*dist*” function in R 4.4.1. A permutational multivariate analysis of variance (PERMANOVA) was then conducted using the “*adonis2*” function from the vegan package (v. 4.0.0) with 999 permutations to evaluate differences in skull parameters among developmental periods. Due to the small sample size in our study, the Kruskal–Wallis test was used to determine the significance of differences in 29 skull parameters of *V. sinensis* across different periods. After the Kruskal–Wallis test, pairwise comparisons were performed using Mann–Whitney U tests with Bonferroni correction to assess the significance of differences in skull parameters across developmental periods, ensuring the reliability and accuracy of the results.

Centroid size is obtained in the GPA process and represents the size of the skull. We compared the centroid size of the skulls of *V. sinensis* from each period. The Mann–Whitney U test was conducted using SPSS 29.0 to determine whether there are significant differences in the centroid size of skulls between different periods.

### 2.4. Comparison of the Skull Space Morphology of V. sinensis at Different Developmental Periods

A chromatogram is commonly used in 3D scanning data to visualize differences or similarities between different regions through color coding. Therefore, to clarify spatial morphological changes during skull development in the *V. sinensis*, comparisons of the spatial morphology of the *V. sinensis* cranium across time were made using Geomagic Control X 2022.0.0 (3D Systems, Inc., Rock Hill, SC, USA). In this analysis, adult *V. sinensis* skulls were used as reference data, and 3D scan data of skulls from each developmental period were used as test data for “Best-fit Align”. Chromatograms were generated by the “3D Comparison” function to visualize the differences and morphological changes among bat skulls. We also used the “*plotRefToTarget*” function in package “geomorph 4.0.8” [45] (R 4.4.1) [46] to perform morphological difference visualization analysis of 3D surface models using the “TPS”, “vector”, and “points” methods.

### 2.5. Comparison of Stretch Factors of V. sinensis Masticatory Muscles at Different Developmental Periods

**Figure 3:**
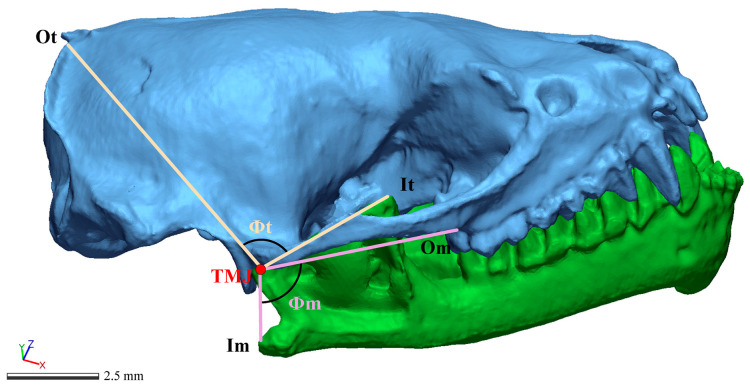
Image of an articulated skull and mandible used to measure the origin (*Ot*) and insertion (*It*) of the temporal muscle, and the origin (*Om*) and insertion (*Im*) of the masseter muscle. The angles between origin and insertion distances for the temporal (*Φt*) and masseter muscles (*Φm*) were drawn.

The SF model describes variation in the placement of the masticatory muscles relative to the temporomandibular joint; changes in the location of muscle origin and insertion relative to the joint may permit wide gapes before the muscle is stretched; the higher the SF, the lower the ability to open the mouth [50]. Differences in masticatory function among *V. sinensis* at different periods were evaluated using the stretching factors of the masseter muscle and temporal muscle. Herring [50] proposed a model for calculating the SF of the superficial masseter muscle and stated that the model is equally suitable for any type of masticatory muscle. Using the model proposed by Herring [50] and the masseter muscle SF measure proposed by Dumont [20], and referring to the temporal muscle SF measure of Sandra [51], the distances between the masseter and temporal muscles at the origin and insertion points on the skull and the angle between the two connecting lines were measured and computed in skull samples of the *V. sinensis* at different developmental periods (Figure 3). The gape capacity is the extent to which the jaws can be opened before the muscle is stretched; it is described by the SF of the masticatory muscles as the ratio between the length of the muscle when the mouth is closed and the length of the muscle when the jaw is rotated through the angle *θ* [50]. In this case, *θ* is equal to 60° [20]. The formula for the masticatory muscle stretching factor is as follows:

(1) Ll2=1−[2ab cos θ+ϕ]/(a2+b2)1−(2ab cosϕ)/(a2+b2)

To minimize errors in SF model construction, we generated three models for each sample. We then calculated the mean SF value for each individual across these repeated measurements. Differences in SF among developmental periods were subsequently assessed using the Mann–Whitney U test on these individual mean values. Additionally, the first six periods of bats were merged to form a subadult bat group. We compared the differences in masticatory muscle SF between adult and subadult bats. Statistical analyses were conducted using SPSS 29.0.

## 3. Results

### 3.1. Traditional Morphometrics

In this study, a total of 21 *V. sinensis* individuals were collected. After determining their ages using the age formula, we measured both field-based morphological parameters and skull parameters on the specimens. The results for each developmental period are presented as mean ± standard deviation (Mean ± SD) in Table S1 and Figures S3 and S4.

**Figure 4:**
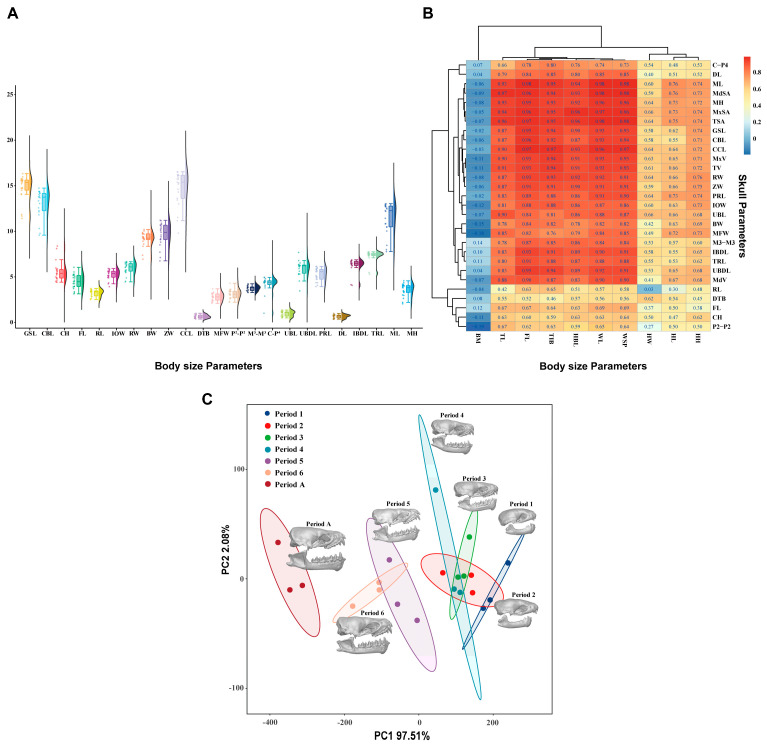
Results of the statistical analysis. (**A**). Basic statistics on the measurements of *V. sinensis* skull parameters. (**B**). Correlation analysis between skull parameters and body size parameters. (**C**). Principal component analysis of skull parameters of *V. sinensis*.

Basic statistical analyses were conducted on the skull measurement parameters of *V. sinensis* (Figure 4A). The correlation analysis of field-obtained *V. sinensis* morphological parameters with measured skull parameters from specimens revealed (Figure 4B) that 24 of the 29 measured skull parameters correlated with the body size parameters of tail length (TL), forearm length (FL), tibia length (TIB), head and body length (HBL), wing length (WL), and wing span (WSP). This result indicates that the overall development of the *V. sinensis* skull follows the developmental dynamics of individual bats, which is basically consistent with the developmental dynamics of body size and shows a high correlation. Principal component analysis (PCA) was performed with SPSS 29.0 based on 29 skull parameters. The KMO (Kaiser–Meyer–Olkin) test was 0.704, which proved that the utility of the data was good [52]. Bartlett’s Test of Sphericity results reject the null hypothesis (χ^2^ = 1193.401, *p* < 0.001), indicating that there is a correlation between the variables, supporting the principal component analysis (PCA) [52]. In the scatter plot based on PC1 and PC2 (Figure 4C), skulls from different developmental periods of *V. sinensis* are separated from each other, with the first four periods clustering together, and Period 5 and Period 6 clustering together. The results show that the first four periods are more similar to each other, while Periods 5 and 6 are clearly different from the first four periods; adults are significantly different from all other periods in terms of distance.

The PERMANOVA results (Table 2) revealed highly significant differences in skull parameters among the different developmental periods (R^2^ = 95.54%, *p* < 0.001). Kruskal–Wallis test analysis revealed that only five measurement parameters: frontal length (FL), rostral length (RL), distance between the tympanic bulla (DTB), distance between P2 (P2-P2), and distance between upper canine and P4 (C-P4), showed no significant differences among developmental periods, while all other parameters exhibited significant differences (*p* < 0.05). Pairwise comparisons with Bonferroni correction revealed significant differences only between Period 1 and other periods; no significant differences were found in skull parameters among other comparison groups (Table S2).

**Figure 5:**
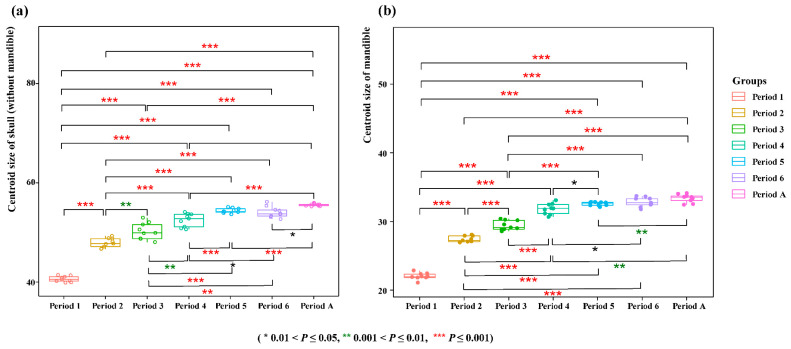
Comparison of centroid size. (**a**) Skull (without mandible) (**b**) Mandible.

Comparisons of skull (without mandible) and mandible centroid sizes revealed that the skull dimensions of *V. sinensis* during Periods 1 to 4 were significantly smaller than those in all subsequent periods. Furthermore, we compared period 1 as an independent period with the other periods after the merger. We found that period 1 had the greatest difference compared to the other periods. (*Z* = −4.773, *p* < 0.001) (Figure 5 and Table S3). These findings align with the results of the statistical analyses.

### 3.2. Geometric Morphometrics

**Figure 6:**
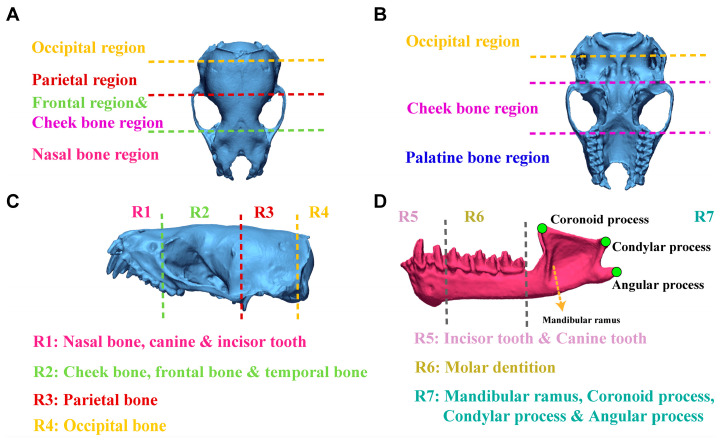
Detailed information on the division of different views of the *V. sinensis* skull using 3D chromatographic analysis. (**A**–**C**) correspond to the dorsal, ventral and lateral views of the skull (without mandible), respectively. The lateral view of the mandible is shown in (**D**).

Based on the anatomical structure of the skull [41,42,43], the dorsal surface of the skull (without mandible) of *V. sinensis* was divided into four regions: occipital region, parietal region, frontal and cheek bone region, and nasal bone region (Figure 6A). The ventral surface of the skull (without mandible) can be divided into four regions: occipital region, cheek bone region, palatine bone region and the dorsal of the parietal bone (Figure 6B). The lateral aspect of the skull (without mandible) is divided into four regions: R1 includes the nasal bones, canines, and incisor teeth; R2 partially includes the cheek bones, frontal bones, and temporal bones; R3 includes the parietal bone; R4 includes the occipital bone (Figure 6C). The lateral view of the mandible is divided into three regions: R5: canine and incisor teeth, R6: molar dentition, R7: mandibular ramus, coronoid process, condylar process and angular process (Figure 6D).

**Figure 7:**
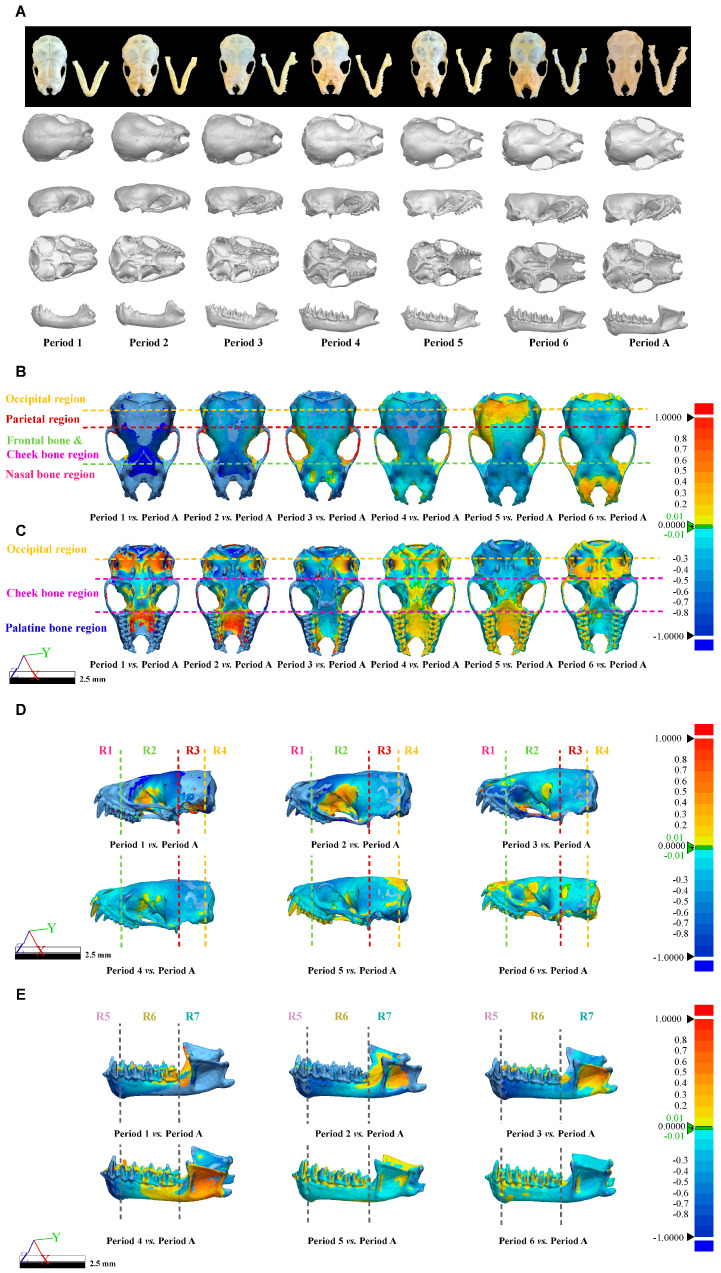
Three-Dimensional Models and Chromatographic analysis of *V. sinensis* Skulls across developmental periods. (**A**). Three-dimensional models of the *V. sinensis* skull for each period in dorsal, lateral, ventral, and lateral mandibular views. Dorsal (**B**), ventral (**C**), and lateral (**D**) views of the skull (without mandible) and lateral (**E**) views of the mandible of the 3D Chromatographic Analysis. The Deviation 3D chromatogram shows the differences in different regions according to a set color mapping, with red being positive difference, blue being negative difference, and green indicating near-zero deviation.

After obtaining the 3D skull structure of *V. sinensis* (Figure 7A), 3D chromato-graphic analysis revealed that from Period 1 to Period 6, the color of the frontal and nasal regions gradually changed from cold to warm colors, demonstrating that the parietal, frontal, and nasal regions gradually changed from raised to smooth from Period 1 to Period 6 compared to Period A. In particular, Period 1 has the darkest chromatogram, and the cranial vault is rounded with the most pronounced convexity (Figure 7B). It is evident from the palatine bone region that the upper buccal dentition of *V. sinensis* in Period 4 is almost fully developed and is essentially the same as that of Period A (Figure 7C). The same result can be obtained from the chromatogram of the lateral view of the skull (without mandible) (Figure 7D); there was almost no difference between R1 and the Period A after Period 4, and the parietal bone and frontal bone gradually changed from cold to warm colors in R2. The lateral view of the mandible of *V. sinensis* shows that (Figure 7E) between period 1 and period 4, the canine and incisor teeth gradually develop fully. The mandibular molar dentition in period 5 is almost indistinguishable from that of Period A. The angular process starts to expand outward from period 1 and becomes fully developed by period 4. Therefore, it can be shown that the Period 1 morphology of *V. sinensis* differs from that of other periods, especially the nasal bone, parietal bone and frontal bone are significantly different compared to other periods, which is consistent with the results of skull parameter statistical analysis (skull parameter discriminant analysis and centroid size difference comparison). In addition, chromatographic analyses revealed that the teeth of *V. sinensis* from Period 4 and Period 5 were essentially fully grown and developed, and the skull phenotype of *V. sinensis* was consistent with that observed when the specimens were obtained in the field (Figure 7A) and that *V. sinensis* in this period was able to fly independently and had completed the transition milk to solid food [35].

**Figure 8:**
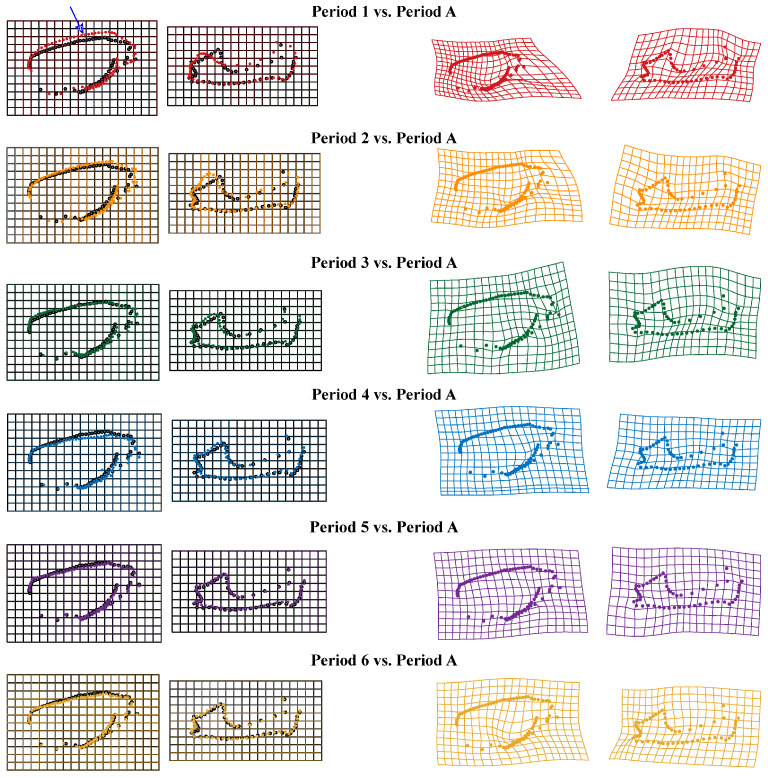
Thin Plate Spline mesh deformation diagram of skull comparison groups from different periods. Arrow 1 indicates the protruding area.

In order to visualize the skull results, the deformation of the Thin Plate Spline (TPS) is plotted by the “*plotRefToTarget*” function. As shown in Figure 8, when using adult bats as a reference, the upper contour line above the facial region of the lateral view of the skull (without mandible) of *V. sinensis* in Period 1 is more prominently protruding than in other comparison groups (marked by arrow 1). Specifically, in Period 1, the parieto-occipital region protrudes outward, while the mandibular angular process is not well defined. We found that the molar dentition of the skull (without mandible) gradually lengthened with growth and development. Canine and incisor teeth grew rapidly during periods 1–3, and the height remains unchanged in Period 4. Meanwhile, the length of the mandibular molar dentition gradually increases, the coronoid process is elevated, the condylar process is gradually lowered and concaves inward, the angular process is expanded and flared, and the structure and outline of the condylar process and angular process gradually become clear. The mandibular ramus is inclined medially and anteriorly, and the distance between the mandibular ramus and the last molar becomes smaller.

### 3.3. Developmental Dynamics of the Stretch Factors of V. sinensis

**Figure 9:**
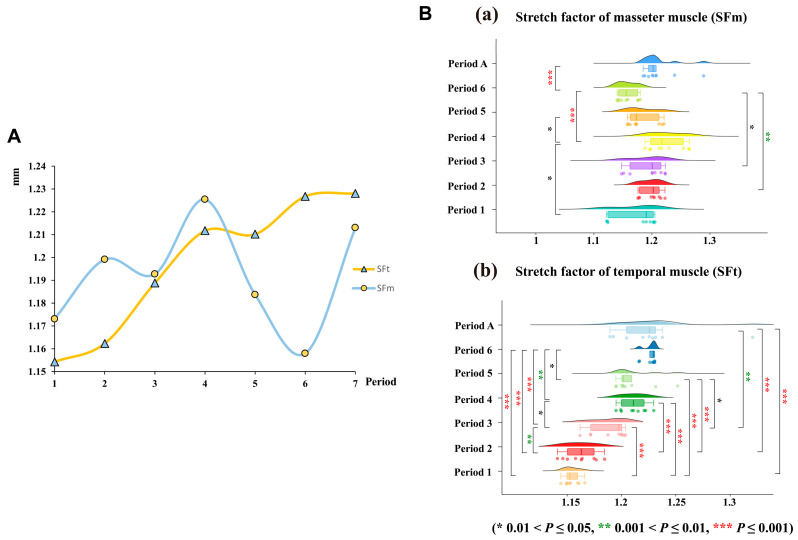
(**A**). Dynamics of change in the SF of the chewing muscle of *V. sinensis* at different developmental periods. (**B**). SF of the masticatory muscle. (**a**) SF of the masseter muscle. (**b**) SF of the temporal muscle.

Our study revealed that the SF of the masseter muscle (SFm) and the temporal muscle (SFt) exhibited dynamic changes throughout the development of *V. sinensis*. During the early developmental periods (Periods 1 to 4), SFm progressively increased, peaking in Period 4, before declining in Periods 5 and 6. SFt followed a similar pattern, decreasing after Period 4 (Figure 9A). The results of the statistical analysis (Mann–Whitney U test) revealed significant differences in the SFt between Periods 1–3 and all subsequent periods (except for Period 1 vs. Period 2). The SFm of Period 6 was a highly significant difference from that of other developmental periods (*p* < 0.001), and it was the lowest during the whole developmental process. Overall, we suggest that Period 4 and Period 6 may be critical periods for both masticatory muscle SF and skull development. In addition, by comparing the differences in SF between adults and subadults, we found that SFt was significantly higher in adults than in subadults (*Z* = 2.495, *p* = 0.013) (Figure 9B and Table S3).

## 4. Discussion

The morphological features of organisms serve as the foundation for taxonomy and are crucial for resolving phylogenetic relationships among fossil taxa and their affinities to living taxa [53]. Variations in the morphology of natural organisms can reflect differences in their functions, developmental processes, and responses to selective pressures [54]. Among all external morphological structures, the skull has been extensively studied as a well-documented nexus of direct interaction between organisms and their environment [29,55,56].

In this study, we examined the developmental dynamics of *V. sinensis* skulls across seven developmental periods, employing a multifaceted approach that included traditional morphometric measurements, geometric morphometric analyses, comparative spatial structural assessments, and SF modeling. Our investigation focused on morphological descriptive parameters, spatial structural variations, and the prediction of opening capacity. Through these comprehensive analyses, we reached the following major conclusions: (1) Synergistic development of skull and body growth. The morphological characteristics of the skull are consistent and strongly correlated with the overall growth and development dynamics of body size in *V. sinensis*. This synergistic development reflects an integrated relationship between skull growth and body growth. While the skull morphology in the first four developmental periods is similar, significant differences emerge in subsequent periods. (2) Ontogeny and adaptation. Three-Dimensional geometric morphometric analysis of *V. sinensis* skulls shows early neonatal traits (e.g., the convex cranial vault in Period 1) are conserved for mammalian brain growth, while subsequent rapid ossification, streamlined cranial contours, and adult-like dentition by Period 4 are key flight adaptations, supporting independent flight and ecological niche occupation. (3) SF and functional adaptation. The SF modeling of the *V. sinensis* skull reveals that the dynamics of its ability to open the mouth align with the trends observed in previous studies on gut microbial community changes. This suggests a functional adaptation that correlates with developmental and ecological shifts.

### 4.1. Synergistic Development Between Skull Morphology and Body Size in V. sinensis

The skull is a key anatomical component of vertebrate organisms, located at the uppermost part of the body [57]. Across diverse species, skull development is often linked to changes in body size during growth. Studies on delayed somatic growth in human children have found that delayed somatic growth affects skull structure development, such as short length of the cranial base and the mandible, increased lower facial height, retropositioned mandible, and obtuse gonion angle [58]. Notably, the association between skull development and body size is not limited to mammals. There are reports indicating that the skull development patterns of non-mammalian species are also influenced by body size development. For instance, the head size in the fish *Chelon auratus* is affected by body size from 24 to 54 days after hatching [59]. Some organisms also have fixed developmental patterns. Among reptiles, snakes and lizards show very similar changes in the relative size of their skulls from juvenile to adult, with both centroid size and linear dimensions doubling during development [60]. The relationship between skull capacity and body size varies among bird species, with over 1400 species exhibiting significant differences in skull capacity that are associated with their specific developmental patterns (e.g., the duration of developmental periods) [61].

However, whether such a correlated, synergistic relationship between skull development and body size in bats remains unclear. Our results indicated that among the 29 measured skull parameters, 24 were positively correlated with body size metrics, including head and body length (HBL), wing length (WL), and forearm length (FL). This indicates that during the postnatal period, from juvenile growth to the onset of independent flight, the overall skull development of *V. sinensis* follows the dynamics of individual morphology, showing a high degree of correlation with body size development. Additionally, forearm length (FL), a key indicator of bat body size [62], is also an important indicator of bat age [63,64], further indicating development across all body parts of *V. sinensis*.

Field observations of *V. sinensis* colonies and previous studies confirm that these bats achieve complete independent flight after Period 4 (28 days post-birth), allowing them to expand their foraging range and access more food resources and suitable roosting sites [35,37]. During the first 28 days, both body weight and forearm length increase linearly, with strong correlations between these traits and age. Consistent with this, our principal component analysis (PCA) of skull measurement parameters showed that the first four developmental periods clustered together with high similarity. We hypothesize that this early-stage similarity is adaptive for bat survival and development, potentially supporting critical functions such as feeding and sensory maturation [65].

### 4.2. Ontogeny and Flight-Adaptive Geometric Changes in the V. sinensis Skull

Bats are the only mammals capable of true flight, and this specialization drives postnatal development of derived phenotypic traits, such as elongated forelimbs, reduced bone cortical thickness, and specialized pectoral girdles, critical for flight efficiency [66,67,68,69]. A key flight-linked adaptation in bat skulls is their rapid ossification: unlike most mammals, bat skulls complete ossification before the onset of independent flight, ensuring structural integrity to withstand aerodynamic forces during flight [70]. And the skull’s rapid ossification provides a stable skeletal framework for the attachment of masticatory muscles (e.g., masseter, temporalis) and the secure anchoring of teeth. For *V. sinensis*, this rapid ossification is not just a “structural prerequisite for flight” but also a “functional foundation for flight-linked foraging behavior”. Our data for *V. sinensis* align with this pattern: we observed accelerated skull development, with the maxillary canine and incisor teeth (key for post-weaning insectivory, a flight-dependent foraging behavior) reaching adult-like morphology by Period 4 (no further obvious deformation thereafter). Concurrently, the convex parietal, frontal, and nasal bone regions of the cranium (prominent in Period 1) gradually smoothed out between Periods 1–6—a change that likely reduces cranial drag and optimizes head aerodynamics, as a streamlined skull is critical for minimizing air resistance during flight.

These observations underscore that while early skull traits (e.g., Period 1 convexity) are linked to general mammalian neonatal development (e.g., brain growth), the subsequent geometric changes (rapid ossification, streamlined cranial contours, and mature dentition by Period 4) are tightly aligned with flight adaptation. As the only volant mammals, bats require skulls that balance light weight (for flight efficiency) and mechanical stability (for foraging and sensory function) [71]. Our results confirm that the spatial structural development of the *V. sinensis* skull is highly optimized to meet these flight-related demands: the transition from a malleable, convex neonatal skull to a streamlined, fully ossified adult skull directly supports the behavioral shift to independent flight—enabling *V. sinensis* to forage over larger ranges and occupy its ecological niche [72].

In addition, to place the skull developmental dynamics of *V. sinensis* in a broader chiropteran evolutionary and ecological context, we compared our findings with data from closely related taxa in the family Vespertilionidae (the same family as *V. sinensis*) and other bat lineages. Consistent with observations in *Myotis myotis* (a congeneric vespertilionid bat [70]), *V. sinensis* exhibits rapid skull ossification prior to the onset of independent flight. This shared trait likely reflects the *V.sinensis* adaptation to flight, as early ossification ensures the bat’s skull can withstand aerodynamic forces while supporting sensory and foraging functions [32,70]. Similarly, like *Noctilio leporinus* (Noctilionidae) [31], newborn *V. sinensis* (Period 1) exhibit a rounded cranial vault. It is a plesiomorphic neonatal feature common to most mammals, serving to accommodate rapid postnatal brain growth. These early characteristics provide the necessary flexibility for subsequent flight-related skull remodeling [31].

### 4.3. Developmental Variations in Stretch Factors of V. sinensis

Numerous functional and comparative studies on mammalian skull morphological development have demonstrated that changes in skull morphology and parameters are closely linked to dietary transitions, particularly during early postnatal development [1]. In the American puma (*Puma concolor*), skull phenotypic shifts align with dietary shifts from milk to solid prey [73], a pattern shared across many mammals. Similarly, postnatal skull development in the white-eared opossum (*Didelphis albiventris*) involves complex modifications to key structures: the palate (a feature critical for milk suckling and later solid food processing [74]), muscle attachment processes (e.g., angular process of the mandible), temporomandibular joint, frontal region, braincase, occipital bones, and petrosal [74]. These modifications highlight the universality of skull adaptation to dietary transitions in mammals.

In bats, skull morphology is also closely linked to diet. Species with different diets exhibit significant differences in skull shape and bite force, and even insectivorous bats show morphological variation (e.g., short, thick skulls for hard-shelled prey vs. long, thin jaws for soft prey) based on food hardness [54,75,76]. For bats with specialized diets (e.g., piscivore bats and nectarivore bats), postnatal skull development is further shaped by the functional demands of transitioning from milk feeding to species-specific feeding modes [31]. Our study found corresponding dynamic changes in masticatory muscle SF during postnatal development of *V. sinensis*. The SF of the masseter muscle (SFm) gradually increased from Period 1 to Period 4 (peaking in Period 4) before declining in Periods 5–6; the SF of the temporal muscle (SFt) also decreased after Period 4. Functionally, SF quantifies the ratio of masticatory muscle length when the jaw is closed to its length when rotated to a 60° gape [20,50]: a higher SF indicates reduced gape capacity but greater potential for force generation (critical for biting hard prey), while a lower SF reflects increased gape flexibility (useful for handling larger prey). This explains the observed SF trends: the peak SFm in Period 4 aligns with the onset of insectivory (needing force to crush insect exoskeletons), and the subsequent decline in Period 6 may reflect an adaptation to more varied prey handling as bats mature.

Beyond dietary pressures, bats face unique selective constraints as the only mammals capable of true flight, constraints that further influence skull developmental trajectories. Flight requires more complex neural control and sensory integration, which in turn modulates skull growth patterns [77]. For instance, in the southeastern myotis *(Myotis austroriparius*) and Brazilian free-tailed bat (*Tadarida brasiliensis*), skull development rate and duration exhibit a significant linear relationship with overall skeletal maturation, reflecting constraints imposed by flight [77]. Over longer time scales, environmental factors like food availability also shape bat skull evolution: the Kuhl’s pipistrelle (*Pipistrellus kuhlii*) showed increased skull size between 1875 and 2007, driven by access to larger, more profitable prey—this shift was accompanied by enhanced bite force, underscoring the link between diet and skull morphology [78]. Our study focuses on the short-term ontogenetic trajectory of *V. sinensis* skulls, and we have found a consistent postnatal increase in skull size mirroring the growth trends of other mammals. Since almost all mammals undergo a dietary transition from milk to solid food, bite force becomes a pivotal factor for food acquisition and processing. Consistent with this, our results reveal that *V. sinensis* skull morphology undergoes notable changes during its critical dietary transition period, integrating both mammalian-wide developmental patterns and bat-specific adaptations.

Notably, the SF trajectory of *V. sinensis* correlates closely with its dietary shift and concurrent changes in gut microbiota. Field observations confirm that *V. sinensis* is weaned and transitions to insectivory by Period 4 [37]; moreover, Yin et al. [35] (studying the same *V. sinensis* population) reported that gut microbial α-diversity increases gradually from Weeks 1 to 4 (matching the rising trend of SFm) and stabilizes in Weeks 5 to 6 (paralleling the post-Period 4 decline in SF). This consistency directly links SF-driven masticatory adaptation to adjustments in the digestive system during the dietary transition, highlighting a coordinated developmental strategy that supports *V. sinensis*’ post-weaning survival and ecological adaptation.

## 5. Conclusions

In this study, we focused on the skull development of *V. sinensis* and employed a comprehensive suite of analytical methods to elucidate the morphological changes, differences in opening capacity, and related influencing factors of the *V. sinensis* skull during growth. Our findings provide a crucial foundation for understanding the growth and development mechanisms of bats. The study reveals that the morphological characteristics of the *V. sinensis* skull are highly consistent and strongly correlated with the overall growth and development dynamics of the body size. The skull’s morphological changes followed a similar dynamic across the first four developmental periods, but significant differences emerged at later periods. Newborn bats in Period 1 exhibited markedly different skull characteristics compared to other periods, due to incomplete ossification, unique morphology, and underdeveloped masticatory and occlusal structures. Comparisons of opening capacity across different periods showed a consistent dynamic with previous studies on gut microbial community changes. From a broader perspective, the developmental changes in the *V. sinensis* skull are a key manifestation of its flight-adapted lifestyle and dietary transitions. These changes not only reflect the species’ biological traits but also provide empirical data for studying the relationship between form and function in biological evolution. Our results offer a vital scientific basis for understanding the growth and development mechanisms of bat skulls and vertebrates in general, with potential applications in ecological conservation and evolutionary biology research.

## Figures and Tables

**Table 1:** Details of the *V. sinensis* sample collection.

Periods	Characters
Period 1: Day 1–Day 7	Young bats are born naked and pink, with tightly closed eyes and folded ears.
Period 2: Day 8–Day 14	The body darkens to a black hue, the eyes open, the ears stand erect, and tiny teeth start to emerge.
Period 3: Day 15–Day 21	Body fur grows, skin color deepens, teeth become sharp, begin to fly clumsily, gradually weaned.
Period 4: Day 22–Day 28	As the body fur grows and the fur color darkens, the teeth become sharp, and the bats are now able to fly freely.
Period 5: Day 29–Day 35	The hair color gradually darkens, and the bats are now fully capable of flying freely.
Period 6: Day 36–Day 42	
Period A: Adult	The hair is blackish brown at the base, with grayish white tips on the back, giving it a grayish brown appearance.

**Table 2:** Results of PERMANOVA of *V. sinensis* skull data.

	*Df*	SumOfSqs	R^2^	F	*Pr* (>F)
Model	6	648,315.5697	0.9554	50.0196	0.001
Residual	14	30,242.8951	0.0446		
Total	20	678,558.4648	1		

## Data Availability

Data are contained within the article and Supplementary Materials. The data presented in this study are available on request from the corresponding author.

## Supplementary Materials

The following supporting information can be downloaded at https://www.mdpi.com/article/10.3390/biology14101389/s1, Figure S1. Dorsal (A), ventral (B), lateral (C) views of the skull (without mandible); Lateral (D) views of mandible displaying craniodental measurements. Figure S2. Landmarks used for skull measurement. (A). Lateral views of the skull (without mandible). (B). Ventral views of skull (without mandible) (C). Mandible. Red dots indicate fixed landmarks and blue dots indicate semi-landmarks on the curves. Table S1. Skull measurements and body size parameters of *V. sinensis* at different developmental periods. (Mean ± SD, mm). Figure S3. Trend chart of skull measurements of *V. sinensis* at different developmental periods. Figure S4. Trend chart of body size parameters of *V. sinensis* at different developmental periods. Table S2. The result of Kruskal–Wallis test. Table S3. The result of the Mann–Whitney U test.

## References

[1] Wainwright P.C. (1991). Ecomorphology: Experimental functional anatomy for ecological problems. Am. Zool..

[2] Arnold S.J. (1992). Constraints on phenotypic evolution. Am. Nat..

[3] Schluter D. (1996). Adaptive radiation along genetic lines of least resistance. Evolution.

[4] Santana S., Dumont E. (2009). Connecting behaviour and performance: The evolution of biting behaviour and bite performance in bats. J. Evol. Biol..

[5] Calsbeek R., Irschick D.J. (2007). The quick and the dead: Correlational selection on morphology, performance, and habitat use in island lizards. Evolution.

[6] Arbour J.H., Curtis A.A., Santana S.E. (2019). Signatures of echolocation and dietary ecology in the adaptive evolution of skull shape in bats. Nat. Commun..

[7] Fostowicz-Frelik Ł., Tseng Z.J. (2023). The mammalian skull: Development, structure and function. Philos. Trans. R. Soc. Lond. B Biol. Sci..

[8] Santana S., Lofgren S. (2013). Does nasal echolocation influence the modularity of the mammal skull?. J. Evol. Biol..

[9] Ikeda Y., Jiang T., Oh H., Csorba G., Motokawa M. (2020). Geographic variations of skull morphology in the *Rhinolophus ferrumequinum* species complex (Mammalia: Chiroptera). Zool. Anz..

[10] Giannini N.P., Wible J.R., Simmons N.B. (2006). On the cranial osteology of chiroptera. I. Pteropus (Megachiroptera: Pteropodidae). Bull. Am. Mus. Nat. Hist..

[11] Davis W.B. (1970). The large fruit bats (genus *Artibeus*) of Middle America, with a review of the *Artibeus jamaicensis* complex. J. Mammal..

[12] Martins F., Ditchfield A., Meyer D., Morgante J.S. (2007). Mitochondrial DNA phylogeography reveals marked population structure in the common vampire bat, *Desmodus rotundus* (Phyllostomidae). J. Zool. Syst. Evol. Res..

[13] Orihuela J. (2011). Skull variation of the vampire bat *Desmodus rotundus* (Chiroptera: Phyllostomidae): Taxonomic implications for the Cuban fossil vampire bat Desmodus puntajudensis. Chiropt. Neotrop..

[14] Kotrschal K., Motta P. (1991). Correlative, experimental, and comparative evolutionary approaches in ecomorphology. Neth. J. Zool..

[15] Korff W.L., Wainwright P.C. (2004). Motor pattern control for increasing crushing force in the striped burrfish (*Chilomycterus schoepfi*). Zoology.

[16] Lappin A.K., Husak J.F. (2005). Weapon performance, not size, determines mating success and potential reproductive output in the collared lizard (*Crotaphytus collaris*). Am. Nat..

[17] Wake D., Roth G. (1990). Evolution and adaptation. (Book reviews: Complex organismal functions. Integration and evolution in vertebrates). Science.

[18] Marroig G., Cheverud J.M. (2004). Did natural selection or genetic drift produce the cranial diversification of neotropical monkeys?. Am. Nat..

[19] Marroig G., Cheverud J.M. (2001). A comparison of phenotypic variation and covariation patterns and the role of phylogeny, ecology, and ontogeny during cranial evolution of New World monkeys. Evolution.

[20] Dumont E., Herrel A., Medellín R., Vargas-Contreras J., Santana S. (2009). Built to bite: Cranial design and function in the wrinkle-faced bat. J. Zool..

[21] Santana S.E., Dumont E.R., Davis J.L. (2010). Mechanics of bite force production and its relationship to diet in bats. Funct. Ecol..

[22] Barlow K.E., Jones G., Barratt E.M. (1997). Can skull morphology be used to predict ecological relationships between bat species? A test using two cryptic species of pipistrelle. Proc. R. Soc. Lond. Ser. B Biol. Sci..

[23] Nogueira M.R., Peracchi A.L., Monteiro L.R. (2009). Morphological correlates of bite force and diet in the skull and mandible of phyllostomid bats. Funct. Ecol..

[24] Pedersen S.C. (1998). Morphometric analysis of the chiropteran skull with regard to mode of echolocation. J. Mammal..

[25] Malhotra A., Thorpe R.S. (1997). Size and shape variation in a Lesser Antillean anole, *Anolis oculatus* (Sauria: Iguanidae) in relation to habitat. Biol. J. Linn. Soc..

[26] Fawcett (1919). The Primordial Cranium of Miniopterus schreibersi at the 17 millimetre Total Length Stage. J. Anat..

[27] Boyd J. (1955). Die Entwicklung und Morphologie des Chondrokraniums von Myotis Kaup. J. Anat..

[28] Camacho J., Heyde A., Bhullar B.A.S., Haelewaters D., Simmons N.B., Abzhanov A. (2019). Peramorphosis, an evolutionary developmental mechanism in neotropical bat skull diversity. Dev. Dyn..

[29] Shi B., Wang Y., Gong L., Chang Y., Liu T., Zhao X., Lin A., Feng J., Jiang T. (2020). Correlation of skull morphology and bite force in a bird-eating bat (*Ia io*; Vespertilionidae). Front. Zool..

[30] Reyes-Amaya N., Jerez A. (2013). Postnatal cranial ontogeny of the common vampire bat *Desmodus rotundus* (Chiroptera: Phyllostomidae). Chiropt. Neotrop..

[31] Monrroy G.A., Reyes-Amaya N., Jerez A. (2020). Postnatal cranial ontogeny of the greater bulldog bat *Noctilio leporinus* (Chiroptera: Noctilionidae). Acta Zoológica.

[32] Santana S.E., Miller K.E. (2016). Extreme postnatal scaling in bat feeding performance: A view of ecomorphology from ontogenetic and macroevolutionary perspectives. Integr. Comp. Biol..

[33] Simmons N.B. (2005). Order Chiroptera. Mammal Species of the World: A Taxonomic and Geographic Reference.

[34] Fukui D., Okazaki K., Miyazaki M., Maeda K. (2010). The effect of roost environment on roost selection by non-reproductive and dispersing Asian parti-coloured bats *Vespertilio sinensis*. Mammal Study.

[35] Yin Z., Sun K., Li A., Sun D., Li Z., Xiao G., Feng J. (2020). Changes in the gut microbiota during Asian particolored bat (*Vespertilio sinensis*) development. PeerJ.

[36] Luo B., Lu G., Chen K., Guo D., Huang X., Liu Y., Feng J. (2017). Social calls honestly signal female competitive ability in Asian particoloured bats. Anim. Behav..

[37] Jin L., Wang J., Zhang Z., Sun K., Kanwal J.S., Feng J. (2012). Postnatal development of morphological and vocal features in Asian particolored bat, *Vespertilio sinensis*. Mamm. Biol..

[38] Jannat N., Islam R., Sultana N. (2023). Preparing and Presenting a Pigeon Skeleton for Gross Anatomical Study Using Boiling Maceration Method: A Quick and Effective Method. Am. J. Life Sci. Innov..

[39] Gofur M., Khan M. (2010). Development of a quick, economic and efficient method for preparation of skeleton of small animals and birds. Int. J. BioRes..

[40] Zhu L., Ma G., Mu Y., Shi R. Reconstruction 3D-models of old Beijing city stuctured light scanning. Proceedings of the 22nd CIPA Symposium.

[41] Bates P.J.J., Harrison D.L. (1997). Bats of the Indian Subcontinent.

[42] Bates P., Thong D., Bumrungsri S. (2005). Voucher Specimen Preparation: Bats.

[43] Yang Q., Xia L., Feng Z. (2007). A guide to the measurement of mammal skull V: Insectivora and Chiroptera. Chin. J. Zool..

[44] Wiley D.F., Amenta N., Alcantara D.A., Ghosh D., Kil Y.J., Delson E., Harcourt-Smith W., Rohlf F.J., St John K., Hamann B. (2005). Evolutionary morphing. Proceedings of the VIS 05. IEEE Visualization.

[45] Adams D.C., Otárola-Castillo E. (2013). Geomorph: An R package for the collection and analysis of geometric morphometric shape data. Methods Ecol. Evol..

[46] R Core Team (2013). R: A Language and Environment for Statistical Computing.

[47] Gower J.C. (1975). Generalized procrustes analysis. Psychometrika.

[48] Rohlf F.J., Slice D. (1990). Extensions of the Procrustes method for the optimal superimposition of landmarks. Syst. Zool..

[49] Webster M., Sheets H.D. (2010). A practical introduction to landmark-based geometric morphometrics. Paleontol. Soc. Pap..

[50] Herring S.W., Herring S.E. (1974). The superficial masseter and gape in mammals. Am. Nat..

[51] Ospina-Garcés S.M., De Luna E., Herrera M. L.G., Flores-Martínez J.J. (2016). Cranial shape and diet variation in *Myotis* species (Chiroptera: Vespertilionidae): Testing the relationship between form and function. Acta Chiropterolo..

[52] Shrestha N. (2021). Factor analysis as a tool for survey analysis. Am. J. Appl. Math. Stat..

[53] Wiens J.J. (2004). The role of morphological data in phylogeny reconstruction. Syst. Biol..

[54] Bonner J.T. (1988). The Evolution of Complexity by Means of Natural Selection.

[55] Jayne B.C., Voris H.K., Ng P.K. (2018). How big is too big? Using crustacean-eating snakes (Homalopsidae) to test how anatomy and behaviour affect prey size and feeding performance. Biol. J. Linn. Soc..

[56] Williams S.H., Peiffer E., Ford S. (2009). Gape and bite force in the rodents Onychomys leucogaster and Peromyscus maniculatus: Does jaw-muscle anatomy predict performance?. J. Morphol..

[57] Anderson B.W., Kortz M.W., Al Kharazi K. (2018). Anatomy, Head and Neck, Skull.

[58] Davidopoulou S., Chatzigianni A. (2017). Craniofacial morphology and dental maturity in children with reduced somatic growth of different aetiology and the effect of growth hormone treatment. Prog. Orthod..

[59] Martinez-Leiva L., Landeira J.M., Fatira E., Díaz-Pérez J., Hernández-León S., Roo J., Tuset V.M. (2023). Energetic implications of morphological changes between fish larval and juvenile stages using geometric morphometrics of body shape. Animals.

[60] Palci A., Lee M.S., Hutchinson M.N. (2016). Patterns of postnatal ontogeny of the skull and lower jaw of snakes as revealed by micro-CT scan data and three-dimensional geometric morphometrics. J. Anat..

[61] Iwaniuk A.N., Nelson J.E. (2003). Developmental differences are correlated with relative brain size in birds: A comparative analysis. Can. J. Zool..

[62] Lindenfors P., Gittleman J.L., Jones K.E. (2007). Sexual size dimorphism in mammals. Sex, Size and Gender Roles: Evolutionary Studies of Sexual Size Dimorphism.

[63] Cheng H., Lee L. (2002). Postnatal growth, age estimation, and sexual maturity in the Formosan leaf-nosed bat (*Hipposideros terasensis*). J. Mammal..

[64] Kunz T.H., Anthony E.L. (1982). Age estimation and post-natal growth in the bat *Myotis lucifugus*. J. Mammal..

[65] Adameyko I., Fried K. (2016). The nervous system orchestrates and integrates craniofacial development: A review. Front. Physiol..

[66] Santana S.E., Grosse I.R., Dumont E.R. (2012). Dietary hardness, loading behavior, and the evolution of skull form in bats. Evolution.

[67] Dickinson M. (2008). Animal locomotion: A new spin on bat flight. Curr. Biol..

[68] Lee A.H., Simons E.L. (2015). Wing bone laminarity is not an adaptation for torsional resistance in bats. PeerJ.

[69] Cubo J., Casinos A. (1998). The variation of the cross-sectional shape in the long bones of birds and mammals. Ann. Sci. Nat.-Zool. Biol. Anim..

[70] Kłys G., Koenig E. (2024). Anatomical and Morphological Structure of the Skull of a Juvenile Specimen of *Myotis myotis* (Chiroptera: Vespertilionidae). Animals.

[71] Thomas S.P., Suthers R.A. (1972). The physiology and energetics of bat flight. J. Exp. Biol..

[72] Dumont E.R. (2007). Feeding mechanisms in bats: Variation within the constraints of flight. Integr. Comp. Biol..

[73] Segura V., Flores D. (2009). Aproximación cualitativa y aspectos funcionales en la ontogenia craneana de Puma concolor (Felidae). Mastozoología Neotrop..

[74] Abdala F., Flores D.A., Giannini N.P. (2001). Postweaning ontogeny of the skull of *Didelphis albiventris*. J. Mammal..

[75] Freeman P.W. (2000). Macroevolution in Microchiroptera: Recoupling morphology and ecology with phylogeny. Evol. Ecol. Res..

[76] Freeman P.W. (1981). Correspondence of food habits and morphology in insectivorous bats. J. Mammal..

[77] Hermanson J.W., Wilkins K.T. (2007). Growth and development of two species of bats in a shared maternity roost. Cells Tissues Organs.

[78] Tomassini A., Colangelo P., Agnelli P., Jones G., Russo D. (2014). Cranial size has increased over 133 years in a common bat, *Pipistrellus kuhlii*: A response to changing climate or urbanization?. J. Biogeogr..

