# Supplementary material for: Novel 3D Scanning and Multi-Angle Analysis Uncover the Ontogenetic Developmental Dynamics of the Skull in Vespertilio sinensis
Source: Biology (Basel). 2025 Oct 11;14(10):1389. doi: 10.3390/biology14101389 (PMC12561190; doi:10.3390/biology14101389)
Supplement: Supplementary file 1 [file biology-14-01389-s001.zip › biology-3818225-supplementary/Supplementary Materials.pdf]

Supplementary Materials

Novel 3D Scanning and Multi-Angle Analysis Uncover the Ontogenetic Developmental Dynamics of the Skull in *Vespertilio sinensis*

Xintong Li [1, 2], Mingyue Bao [1, 2], Yang Chang [4], Hui Wang [1, 2, *], Jiang Feng [1, 2, 3, *]

[1] College of Life Science, Jilin Agricultural University, Changchun 130118, China.

[2] Jilin Provincial International Cooperation Key Laboratory for Biological Control of Agricultural Pests, Changchun 130118, China.

[3] Jilin Provincial Key Laboratory of Animal Resource Conservation and Utilization, Northeast Normal University, Changchun 130117, China.

[4] College of Life Science, Northeast Agricultural University, Harbin 150030, China.

* Corresponding authors: wangh681@nenu.edu.cn (H.W.); fengj@nenu.edu.cn (J.F.)

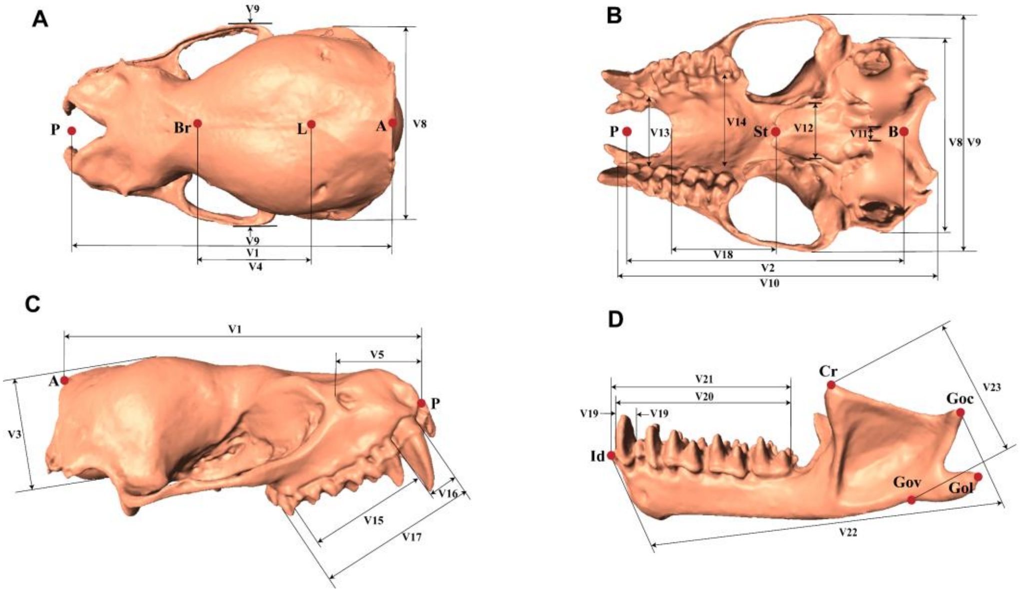

Figure S1. Dorsal (A), ventral (B), lateral (C) views of the skull (without mandible); Lateral (D) views of mandible displaying craniodental measurements.

Detailed descriptions of each metric are：

P (Prosthion): The midpoint at the very front end of both anterior jawbones. Because of the front ends of the two premaxilla of *V.sinensis* are separated, the midpoint of the line connecting the front ends of the two premaxilla is adopted.

A (Akrokranion): The protrusion at the last edge of the top of the skull.

B (Basion): The midpoint of the lower edge of the foramen magnum.

Br: The starting point of the frontal bone suture.

L: The endpoint of the frontal bone suture.

St (Staphylion): The intersection point of the anterior edge of the interpterygeal foramen and the palatine suture.

Id (Infradentale): The midpoint at the very front end of the mandible. That is, the base of the alveolar groove on the outer edge of the middle slit of the incisors.

Cr (Coronion): The apex of the upper edge of the mandibular coronal process.

Goc (Gonion caudale): The distal point of the posterior margin of the articular process.

Gov (Gonion ventral): The lowest point at the bottom of the angular process.

Gol (Gonion laterale): The last margin of the ceratoid process is further away.

Detailed descriptions of each metric:

V1: Greatest skull length (GSL): Distance from point P to A; the maximum length of the skull, the straight-line distance from the most anterior part of the skull (the most anterior projection, including the incisors) to the most posterior projection.

V2: Condylo-basal length (CBL): Distance from point P to B; the straight-line distance from the outer edge of the occipital condyle to the anterior end of the canine teeth.

V3: Cranial height (CH): Straight-line distance from the cranial apex to the lowest point.

V4: Frontal length (FL): Distance from point Br to L.

V5: Rostral length (RL): Distance from the most anterior part of the skull to the last margin of the preorbital foramen.

V6: Interorbital width (IOW): Minimum width between the two preorbital foramina of the muzzle.

V7: Rostral width (RW): Maximum width between the two preorbital foramina of the muzzle.

V8: Braincase width (BW): The distance between the vertices of the outermost margins of the two sides of the skull (without mandible), i.e., the maximum width of the skull (without mandible).

V9: Zygomatic width (ZW): Distance between the most prominent points of the 2 outer edges of the zygomatic arches.

V10: Condylo-canine length (CCL): The distance from the outer edge of the occipital condyle to the anterior end of the canine sulcus.

V11: Distance between the tympanic bulla (DTB): The minimum distance between the inner edges of the two auditory vesicles.

V12: Mesopterygoid fossa width (MFW): Maximum width between the inner edges of the mesopterygoid fossae.

V13: Distance between P2 (P2-P2): Minimum distance between the inner edges of the alveoli of the two skull (without mandible) second premolars (P2).

V14: Distance between M3 (M3-M3): Minimum distance between the inner margins of the alveoli of the two skull (without mandible) third molars (M3).

V15: Distance between upper canine and P4 (C-P4): Distance from the posterior edge

of the upper canine (C) alveolus to the anterior edge of the fourth premolar (P4) alveolus.

V16: Upper canine base length (UBL): Maximum length of the base of the upper canine. The distance from the most anterior to the most posterior edge of the upper canine alveolus.

V17: Upper buccal dentition base length (canine included) (UBDL): Distance from the most anterior margin of the skull (without mandible) canine alveolus to the point of the most posterior margin of the last molar alveolus.

V18: Palatal bridge length (PRL): The distance from the point of the final margin of the unilateral incisor foramen to the point of the most anterior margin of the ipsilateral mesopterygoid fossa.

V19: Diastema length (DL): Distance from the most anterior margin of the third incisor alveolus to the most anterior margin of the premolar alveolus.

V20: Inferior buccal dentition base length (canine included) (IBDL): Distance from the most anterior margin of the mandibular canine alveolus to the point of the most posterior margin of the last molar alveolus.

V21: Tooth row length (TRL): Distance from point Id to the last margin of the last molar sulcus.

V22: Mandible length (ML): Distance from point Id to Goc; maximum length from the anterior end of the mandible to the end of the skull (without mandible) symphysis.

V23: Mandible height (MH): Distance from point Cr to Gov; the vertical distance of the mandible from the upper edge of the mandible (usually the horizontal line of the mandibular ramus or mandibular angle) to the lower edge of the mandible (usually the anterior or posterior edge of the mandibular body).

V24: Skull (without mandible) surface area (MxSA): Total skull (without mandible) surface area.

V25: Skull (without mandible) volume (MxV): Total skull (without mandible) volume.

V26: Mandibular surface area (MdSA): Overall mandibular surface area.

V27: Mandibular volume (MdV): Overall mandibular volume.

V28: Total surface area (TSA): The sum of the surface area of the skull (without

mandible) plus the mandible.

V29: Total volume (TV): The sum of the volume of the maxilla plus the mandible.

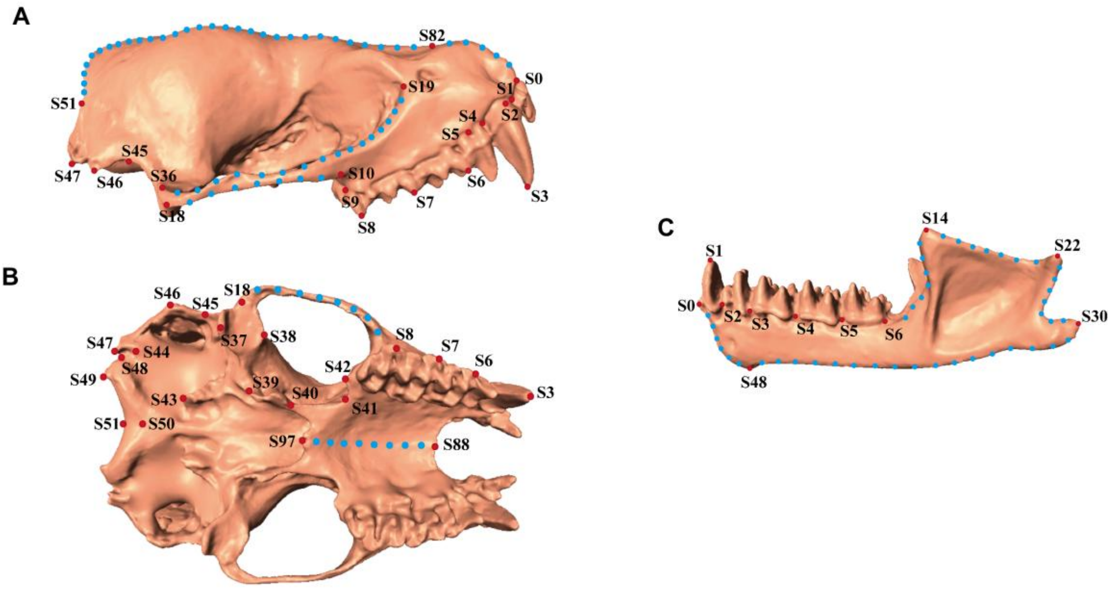

Figure S2. Landmarks used for skull measurement. (A). Lateral views of skull (without mandible). (B). Ventral views of skull (without mandible) (C). Mandible. Red dots indicate fixed landmarks and blue dots indicate semi-landmarks on the curves.

Description of landmarks used in geometric morphometrics of skull (without mandible):

S0: The leading edge point of the upper incisors.

S1: The posterior margin point of the upper incisors.

S2: Leading edge point of canine teeth.

S3: Canine tooth endpoints.

S4: The posterior margin point of the canine teeth.

S5: The very front end of the molar dentition.

S6: The end point of the first molar.

S7: The end point of the second molar.

S8: The end point of the third molar.

S9: The outer edge point of the third molar.

S10-S18: Observe the semi-marker point of the zygomatic arch from the abdominal side.

S19: The very front point of the eye socket.

S20-S35: Observe the half-mark point of the zygomatic arch on the back.

S36: The intersection point of the posterior zygomatic arch and the skull (without mandible).

S37: The mandibular fossa protrudes backward.

S38: Midpoint of the edge of the mandibular fossa.

S39: The anterior endpoint of the cochlea.

S40: The lowest point on the ventral side of the winglet hook.

S41: The endpoints of the tempororfrontal joint.

S42: The posterior endpoint of the third molar.

S43: The cochlea is the endpoint near the center of the body.

S44: The posterior endpoint of the cochlea.

S45: The most dorsal point on the outer edge of the cochlea.

S46: Mastoid process.

S47: Lateral malleolus.

S48: The lateral endpoints of the occipital-ankle.

S49: The final endpoint of the occipital ankle.

S50: The anterior endpoint of the foramen magnum.

S51: The midpoint of the dorsal side of the foramen magnum.

S52-S82: View the upper contour line from the side of the skull (without mandible).

S83-S87: Nasal bone contour line.

S88: Skull (without mandible) incision.

S89-S96: Midpalatal suture.

S97: Sphenopalatine incision.

Description of landmarks used in geometric morphometrics of mandible:

S0: The tip of the mandibular union.

S1: The tip of the lower canine tooth.

S2-S3: Premolar row.

S3-S6: Molar row.

S7-S14: The contour between the ceratoid process and the coronal process.

S14: The tip of the coronal process.

S22: The tip of the ankle process

S30: The tip of the angular process.

S7-S29: Mandibular process contour.

S31-S47: The contour of the lower jawline.

S48: The junction of the mandibular dentary bone.

S49-S54: The contour between the junction of the dentary and the midpoint of the incisors.

Table S1. Skull measurements and body size parameters of *V. sinensis* at different developmental periods. (Mean±SD, mm).

	Period 1	Period 2	Period 3	Period 4	Period 5	Period 6	Period A
**Skull parameters**							
GSL	11.62±0.2166	14.2667±0.3754	14.8667±0.4332	15.25±0.4979	15.7467±0.2001	15.4867±0.2676	15.8367±0.4082
CBL	9.8667±0.3787	12.05±0.233	13.2333±0.8045	14.0533±0.2996	14.1133±0.5278	14.0133±0.5918	13.9767±0.3156
CH	4.71±0.26	4.5733±0.2194	4.99±0.3704	5.4567±0.3459	5.6367±0.2859	6.62±1.5162	6.86±1.23
FL	3.6467±0.8355	3.6433±0.2695	4.4167±0.4709	5.2067±0.3101	4.5833±0.4188	4.8133±0.7569	5.1367±0.895
RL	2.5533±0.0603	3.2567±0.41	3.36±0.2193	3.55±0.1552	3.2567±0.4092	3.2667±0.1888	3.2167±0.2754
IOW	4.15±0.2138	5.0433±0.0961	5.1433±0.265	5.32±0.3639	5.4267±0.3099	5.6633±0.2854	5.75±0.15
RW	4.6±0.2364	5.5967±0.1405	5.7333±0.295	6.09±0.3223	6.2833±0.2376	6.4433±0.2442	6.5933±0.0153
BW	7.54±0.7269	9.12±0.0866	9.07±0.4349	9.4367±0.4735	9.63±0.2987	9.36±0.2606	9.8567±0.4196
ZW	7.1767±0.4796	8.8867±0.4528	9.37±0.4267	9.92±0.7968	10.2367±0.5766	10.4467±0.363	11.0133±0.2639
CCL	10.81±0.909	13.33±0.3079	14.7533±0.5561	15.4467±0.3894	16.01±0.2787	16.1167±0.3755	16.2267±0.2479
DTB	0.5133±0.0862	0.47±0.1587	0.54±0.13	0.6567±0.1986	0.58±0.1253	0.8967±0.1069	0.8033±0.1193
MFW	2.2±0.1493	2.4267±0.2779	2.5533±0.1528	3.0833±0.2074	2.9533±0.1986	3.0733±0.2468	3.21±0.4133
P2-P2	2.64±0.4214	2.8033±0.4464	2.7367±0.2155	3.32±0.2851	3.5967±0.607	3.1733±0.4579	3.47±0.1136
M3-M3	-	3.425±0.2333	3.5267±0.293	3.7067±0.2003	3.8933±0.351	3.7967±0.0874	4.01±0.2007
C-P4	-	3.8133±0.9657	4.21±0.2287	3.39±2.0787	4.5967±0.2822	4.4433±0.1041	4.4867±0.4262
UBL	0.6167±0.0651	0.52±0.0361	0.7767±0.0666	1.1233±0.0577	1.17±0.1323	1.18±0.0557	1.15±0.1769
UBDL	-	4.42±0.9299	5.3933±0.1793	6.2133±0.1301	6.6867±0.8981	6.2033±0.0208	6.4267±0.2994
PRL	4.1033±0.215	4.6667±0.2902	5.1367±0.41	5.31±0.5384	5.81±0.2261	5.9067±0.1935	5.8367±0.2495
DL	0.4±0.03	0.3833±0.0751	0.66±0.0656	0.85±0.1044	0.7467±0.1305	0.71±0.0173	0.8033±0.0404
IBDL	-	4.09±0.0566	6.17±0.1609	6.35±0.1253	6.59±0.2287	6.6333±0.1457	6.67±0.1015
TRL	-	5.375±0.1202	7.26±0.1808	7.4667±0.1504	7.46±0.0346	7.5633±0.2325	7.6±0.0755
ML	8.0567±0.3482	10.3033±0.2548	11.01±0.3466	12.39±0.3223	12.6167±0.1595	12.7±0.1752	12.95±0.0721

MH	2.2767±0.3066	3.0967±0.2203	3.3733±0.1002	3.92±0.1127	4.0633±0.1106	3.98±0.1609	4.2867±0.2875
MxSA	309.2716±10.1983	404.1792±26.2203	439.4888±14.5172	502.1646±12.5064	519.669±20.0965	513.4865±22.649	548.4871±23.3756
MxV	187.6895±25.02	276.849±10.3566	309.7865±30.4537	335.9796±41.8902	354.4974±5.7963	363.2608±21.3316	397.9005±3.7677
MdSA	78.8516±4.63	118.5422±4.4374	138.7197±4.7541	180.9477±19.4866	184.8367±3.9218	181.3979±6.8187	199.0005±10.9627
MdV	22.3537±1.3221	34.334±1.2964	35.12±1.4789	41.0478±4.5119	40.4811±2.3815	38.1524±3.3959	44.8331±3.7322
TSA	388.1232±14.5445	522.7214±30.5449	578.2085±14.4634	683.1123±8.4385	704.5057±16.1834	694.8844±26.4549	747.4875±32.3621
TV	210.0433±26.3244	311.1829±10.9233	344.9065±31.8777	377.0274±44.6755	394.9785±8.1547	401.4132±22.9378	442.7336±1.2904
**Body size parameters**							
Forearm length	17.4633±1.7137	31.67±0.5384	39.5633±0.4899	47.1133±0.2627	49.4567±0.8532	48.79±0.4073	49.2067±0.4708
Body mass	3.87±0.6115	8.9567±0.9744	10.3733±1.4922	12.17±1.016	16.0967±1.0971	15.8233±1.9894	17.9067±0.7151
Head length	15.2467±0.5393	18.2267±0.1563	15.37±4.3367	20.0367±0.3443	20.4167±0.7751	20.6967±0.4325	21.8467±0.9142
Head width	8.86±0.3027	9.26±0.8707	10.035±0.9546	9.0667±0.8003	11.9967±1.1178	12.1433±0.3232	11.2967±1.3984
Head height	6.71±0.2291	7.79±0.7709	7.6367±0.925	8.1167±0.1779	8.84±0.1345	9.01±0.1136	8.3667±1.064
Head and body length	37.4833±2.1865	46.0767±2.7258	50.6433±2.8008	54.7067±2.3152	61.38±3.0939	60.5967±2.2993	61.48±2.5548
Tibia length	7.9867±1.3725	11.88±0.8551	15.1933±0.1823	16.28±0.466	17.37±1.0933	17.1867±0.7868	18.2667±0.525
Tail length	14.7133±0.4102	22.01±1.7806	25.5633±0.4532	37.3067±1.7321	42.7633±4.3002	36.31±0.9732	45.4067±3.755
Wing length	46.9667±3.3872	80.17±3.1298	103.9067±4.7471	133.7333±5.5559	141.9067±3.2038	138.7367±3.3055	146.0167±2.9777
Wing span	56.5367±5.0681	92.2167±3.3354	122.99±6.2664	152.0567±9.0598	161.2033±1.6151	157.0033±3.7495	168.9133±3.418

Skull parameters

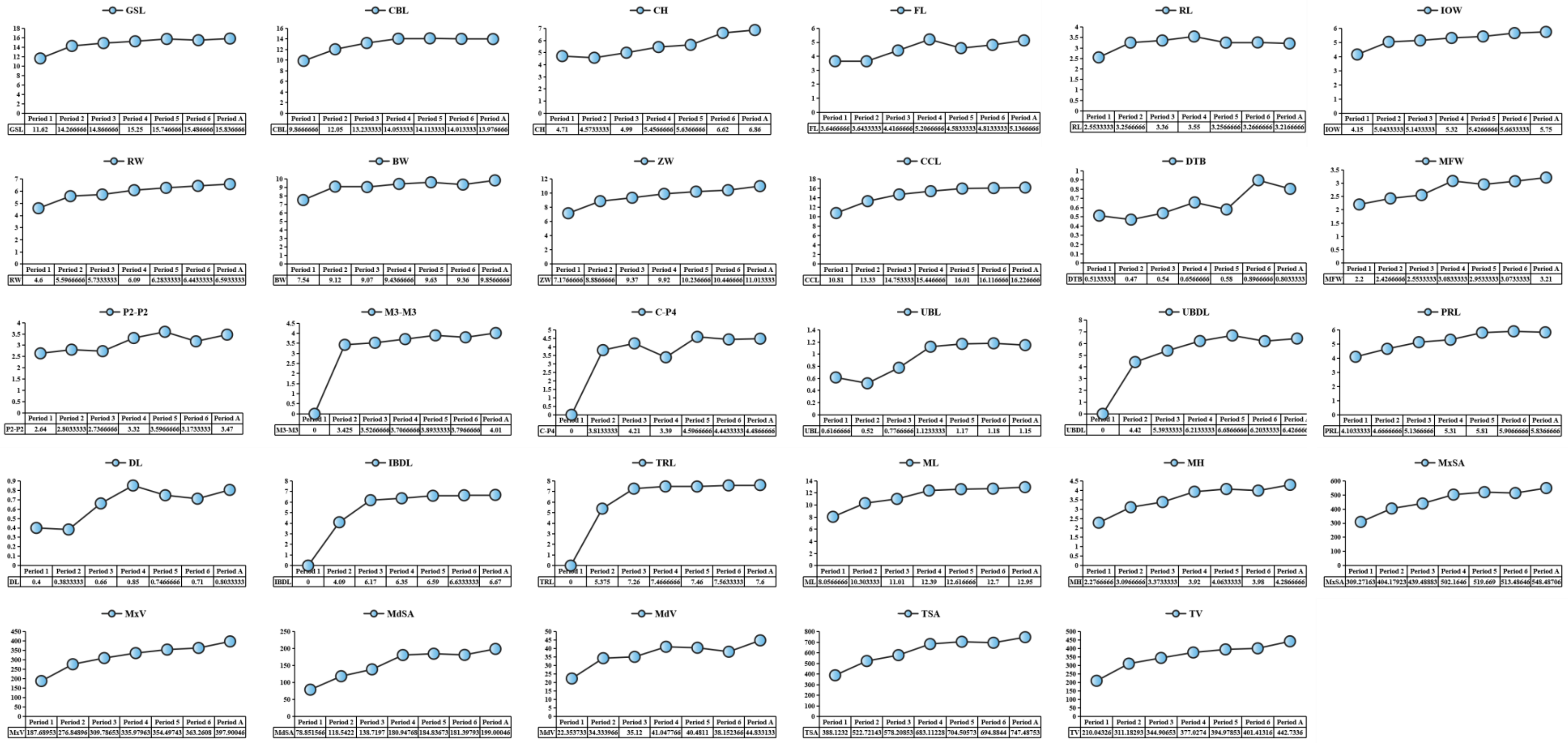

Figure S3. Trend chart of skull measurements of *V. sinensis* at different developmental periods.

Body size parameters

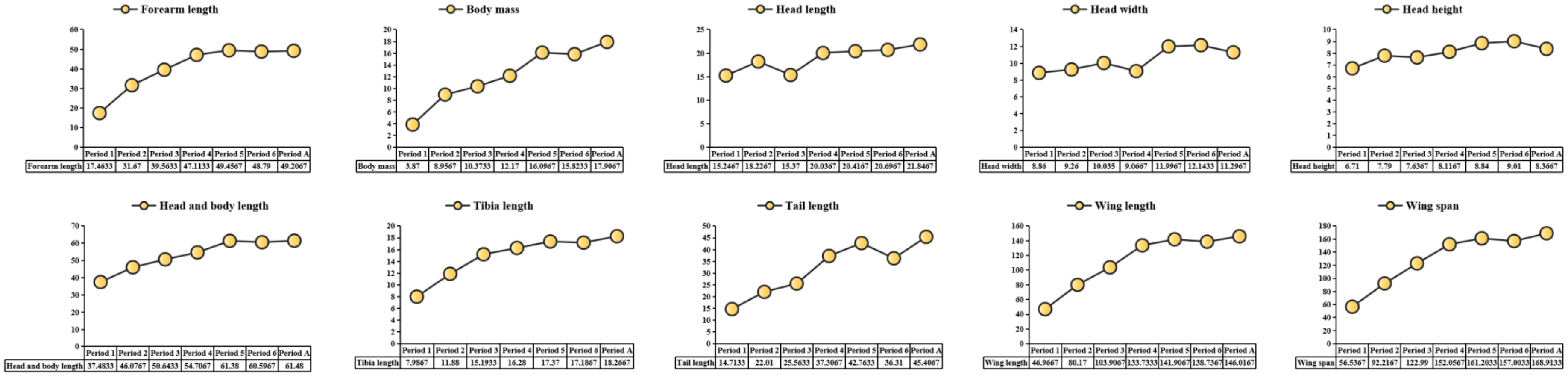

Figure S4. Trend chart of body size parameters of *V. sinensis* at different developmental periods.
